# Prevalence of 16S rRNA Methylation Enzyme Gene *armA* in *Salmonella* From Outpatients and Food

**DOI:** 10.3389/fmicb.2021.663210

**Published:** 2021-05-25

**Authors:** Xin Lu, Mei Zeng, Ning Zhang, Mengyu Wang, Baoke Gu, Jiaqi Li, Huiming Jin, Wenjia Xiao, Zhe Li, Hongqun Zhao, Haijian Zhou, Zhenpeng Li, Jialiang Xu, Xuebin Xu, Biao Kan

**Affiliations:** ^1^State Key Laboratory of Infectious Disease Prevention and Control, National Institute for Communicable Disease Control and Prevention, Chinese Center for Disease Control and Prevention, Beijing, China; ^2^Beijing Advanced Innovation Center for Food Nutrition and Human Health, Beijing Technology and Business University, Beijing, China; ^3^Children’s Hospital of Fudan University, Shanghai, China; ^4^School of Light Industry, Beijing Technology and Business University, Beijing, China; ^5^Jiangxi Provincial Key Laboratory of Preventive Medicine, School of Public Health, Nanchang University, Nanchang, China; ^6^Department of Microbiology, Shanghai Municipal Center for Disease Control and Prevention, Shanghai, China

**Keywords:** *Salmonella*, *armA*, multi-drug resistant, poultry, swine

## Abstract

*Salmonella* is the primary cause of community-acquired foodborne infections, so its resistance to antimicrobials, such as aminoglycosides, is a public health issue. Of concern, aminoglycoside resistance in *Salmonella* is increasing rapidly. Here, we performed a retrospective study evaluating the prevalence of *Salmonella* harboring *armA*-mediated aminoglycoside resistance in community-acquired infections and in food or environmental sources. The prevalence rates of *armA*-harboring *Salmonella* strains were 1.1/1,000 (13/12,095) and 8.7/1,000 (32/3,687) in outpatient and food/environmental isolates, respectively. All the *armA*-harboring *Salmonella* strains were resistant to multiple drugs, including fluoroquinolone and/or extended-spectrum cephalosporins, and most (34/45) belonged to serovar Indiana. The *armA* gene of these strains were all carried on plasmids, which spanned five replicon types with IncHI2 being the dominant plasmid type. All the *armA*-carrying plasmids were transferable into *Escherichia coli* and *Acinetobacter baumannii* recipients. The conjugation experiment results revealed that the *armA*-harboring *S*. Indiana strains had a relatively higher ability to acquire *armA*-carrying plasmids. The low similarity of their pulsed field gel electrophoresis patterns indicates that the *armA*-harboring *Salmonella* strains were unlikely to have originated from a single epidemic clone, suggesting broad *armA* spread. Furthermore, the genetic backgrounds of *armA*-harboring *Salmonella* strains isolated from outpatients exhibited higher similarity to those isolated from poultry than to those isolated from swine, suggesting that poultry consumption maybe an infection source. These findings highlight an urgent need to monitor the prevalence and transmission of *armA*-harboring *Salmonella*, especially *S.* Indiana, to better understand the potential public health threat and prevent the further spread of these strains.

## Introduction

Aminoglycoside antibiotics are widely used in hospitals for the treatment of infections, especially those caused by Gram-negative bacteria (Doi et al., 2004), and can be used synergistically with many other antibiotics (Xia et al., 2016). Aminoglycosides were also used extensively in food-producing animals to prevent bacterial infections (Chen et al., 2007); several clinical first-line aminoglycoside agents were misused in conventional broiler chicken and swine production facilities for the prevention of infections, as well as for growth promotion (Qin et al., 2012; Xia et al., 2016).

Bacterial resistance to aminoglycosides was initially found to be due to the production of aminoglycoside-modifying enzymes, including acetyltransferases, phosphotransferases, and nucleotidyltransferases, which may modify aminoglycosides into non-functional forms (Ramirez and Tolmasky, 2010). 16S rRNA methylation enzymes were thought to be confined to environmental strains until the discovery of *armA* gene-mediated aminoglycoside resistance in *Klebsiella pneumoniae* in 2003 (Galimand et al., 2003). To date, ten different 16S rRNA methylase genes, *armA*, *rmtA*, *rmtB*, *rmtC*, *rmtD*, *rmtE*, *rmtF*, *rmtG*, *rmtH*, and *npmA*, have been reported in various Enterobacteriaceae strains, and the 16S rRNA methylases encoded by these genes confer high-level resistance to various aminoglycosides (Galimand et al., 2012; Wachino and Arakawa, 2012; Bueno et al., 2013; O’Hara et al., 2013). Because the 16S rRNA methylase genes are located on plasmids, they can be easily transferred to other bacteria (Wachino et al., 2007). Therefore, these genes should be monitored by surveillance as a public health precaution.

*Salmonella* spp., which are members of the family Enterobacteriaceae, cause a broad range of diseases in humans, mainly diarrhea and systemic infections, and are one of the greatest food safety threats (LaRock et al., 2015). These bacteria usually live in the intestinal tracts of various animals and humans and can be excreted into the environment via feces (Michael and Schwarz, 2016). *Salmonella* spp. often exhibit resistance to multiple antibiotics and are recognized as very important bacterial vectors of drug-resistance genes in the spread of antibiotic resistance (Crump et al., 2015; Kariuki et al., 2015; Wen et al., 2017). In several aminoglycoside-resistant Enterobacteriaceae (Ma et al., 2009), including *Salmonella* (Naas et al., 2011; Du et al., 2012), the most common 16S rRNA methylase gene is *armA*. Since the report of its discovery in *K. pneumoniae* strains isolated from patients in 2003 (Galimand et al., 2003), plasmids containing *armA* have been detected in diverse bacterial species (Du et al., 2012; Wang et al., 2017b; Uechi et al., 2018). The plasmids carrying *rmtB* were found in S. Typhimurium isolated from food-animal products, and found could co-spread with qepA and blaCTX-M-27 in *S.* Indiana strains from water husbandry (Fang et al., 2019). In addition, other 16S rRNA methylase genes such as *rmtC* and *rmtD* were observed in *Salmonella enterica*, *S.* Indiana and *S.* California strains from chicken samples or people, but are less common (Folster et al., 2009; Hopkins et al., 2010; Wang et al., 2017c). The other 16S rRNA methylase genes such as *rmtE*, *rmtF*, *rmtG*, and *rmtH* have never been reported in *Salmonella* strains.

However, the epidemic and transmission trends of this gene in *Salmonella* are less defined. Plasmids carrying *armA* may allow easy transmission of this gene among various bacterial species, including pathogenic strains.

The prevalence of *Salmonella* harboring plasmids containing *armA* needs to be monitored in people, food, and environmental sources. Here, we conducted a retrospective study to characterize the epidemic status and transmission of *armA* in *Salmonella* strains isolated from outpatients as well as from food and environmental sources.

## Materials and Methods

### Bacterial Strains

Beginning in 2005, *Salmonella* infections in diarrheal outpatients were surveyed yearly in Shanghai. Following the expansion of the participating surveillance laboratories in June 2010, the annual number of isolated strains increased from 2011. Of the 15,782 *Salmonella* strains used in this study: 12,095 strains were collected from outpatient stools in Shanghai between 2005 and 2016; 3,206 strains were collected from various food or environmental samples, including chicken, pork, water, and seafood, in Shanghai between 2009 and 2015; and 481 strains were collected from various food or environmental samples, including chicken, pork, water, and seafood, in Guangdong between 2013 and 2015. All strains were recovered from the strain pool and isolated in CHROMagar *Salmonella* agar (CHROMagar Company, Paris, France). Suspected isolates were identified with the Vitek-II system (BioMerieux, Lyon, France).

### Resistance Gene Amplification

All *Salmonella* isolates were tested for the presence of the *armA* gene by using real-time polymerase chain reaction (PCR) (Supplementary Table 1). All *armA*-harboring Salmonella strains were then subjected to additional PCR assays to assess the presence of aminoglycoside-resistant genes (*ant(2”)-Ia*, *aph(3′)-Ia*, *aac(3)-Ia*, *aac(3)-IIa*, *aac(6′)-Ib*, *rmtA*, *rmtB*, *rmtC*, *rmtD*, *rmtE*, *rmtF*, *rmtG*, *rmtH*, and *npmA*), extended spectrum β-lactamase (ESBL)/AmpC genes (*bla*_DHA_, *bla*_TEM_, *bla*_CMY_, *bla*_OXA_, *bla*_CTX–M_, and *bla*_SHV_), plasmid-mediated quinolone resistance (PMQR) genes (*qnrA*, *qnrB*, *qnrC*, *qnrD*, *qnrS*, *oqxA*, *oqxB*, and *qepA*), the colistin-resistant gene *mcr-1*, and the carbapenem-resistant gene bla_NDM_. Primers (Wu et al., 2009; Gopalakrishnan et al., 2018; Taylor et al., 2018) used in this study were showed in Supplementary Table 1. The variants of *bla*_CTX–M_ were confirmed by Sanger sequencing. The PCR amplification products were also sequenced, and the resulting DNA sequences were analyzed by using the BLAST program^1^.

### Antimicrobial Susceptibility Testing

Antimicrobial susceptibility testing was performed on all the *armA*-positive isolates using the reference broth microdilution method with custom plates (PRCDCN2, Thermo) (Clinical and Laboratory Standards Institute [CLSI], 2015) for the following 20 antibiotics: ampicillin, tetracycline, erythromycin, chloramphenicol, cefazolin, ciprofloxacin, trimethoprim/sulfamethoxazole, ceftazidime, imipenem, nalidixic acid, cefoxitin, cefotaxime, gentamicin, azithromycin, ceftazidime, amikacin, tobramycin, cefepime, colistin, and tigecycline. The results were assessed with the CLSI (2015) breakpoints (Clinical and Laboratory Standards Institute [CLSI], 2015). The control strain is ATCC 25922.

### Pulsed Field Gel Electrophoresis (PFGE) Analysis

All *armA*-harboring *Salmonella* strains were analyzed by pulsed field gel electrophoresis (PFGE) to determine their genetic homology. Bacterial DNA was digested with the restriction enzyme *Xba*I and then analyzed by electrophoresis for 19 h at 6 V/cm, with a pulse angle of 120°, a temperature of 14°C, and pulse times ranging from 2.16 to 63.8 s. Comparison of the resulting PFGE patterns was performed with Bionumerics software (Applied Maths, Sint-Martens-Latem, Belgium) based on the Tenover’s criteria (Tenover et al., 1995). Isolates were allocated into genetic similarity clusters using a cut-off value of 80%.

### Plasmid and Southern Blot Analyses

To detect the sizes of the *armA*-carrying plasmids, agarose gel plugs containing total cellular DNA were prepared and digested with S1 nuclease (TaKaRa, Dalian, China) as described previously (Barton et al., 1995). Digested plugs were subjected to PFGE using a CHEF-Mapper system (pulse times, 2.16–63.8 s; running time, 19 h; 6 V/cm). Gels were blotted onto nylon membranes (Millipore, United States). The membranes were hybridized with a digoxigenin-labeled probe directed against *armA*.

Based on the Southern hybridization results, the gel slices resulting from PFGE following digestion with S1 nuclease found to contain the *armA* fragment were excised. The DNA fragments from the gels were purified and used as templates for identifying the plasmid replicon type by PCR. A PBRT kit (Diatheva, Italy) was applied for plasmid molecular typing (Garcia-Fernandez et al., 2009).

### Plasmid Elimination Test

For the SDS treatment method, 50 μl of *armA*-positive *Salmonella* suspension (OD = 0.5–0.7) was inoculated into 5 ml of LB medium with 10% SDS and then incubated in a thermostatic shaker at 37°C for 18–24 h; an SDS concentration one-half of that in which growth was first observed was chosen as the working concentration for the elimination test (Wangkheimayum et al., 2017). The products were diluted ten times with saline and then selected on agar plates containing 1000 μg/ml amikacin and on plates without antibiotics. The plasmid-eliminated strains were identified by real-time PCR.

### Plasmid Conjugation

The conjugation test was performed with the broth mating method using all 45 aminoglycoside-resistant *Salmonella* strains as the donor strains and sodium azide-resistant *Escherichia coli* J53 or streptomycin-resistant *Acinetobacter baumannii* as the recipient strains. The transconjugants were selected on agar plates containing 30 μg/ml amikacin and 400 μg/ml sodium azide when using *E. coli* J53 as the recipient and on LB agar plates containing 30 μg/ml amikacin and 5,000 μg/ml streptomycin when using *A. baumannii* as the recipient. The resulting transconjugants were identified by real-time PCR targeting the *armA* gene. The transfer frequency is expressed as the number of transconjugants per total recipients.

To test the ability of the *Salmonella* strains from different serotypes to obtain an *armA*-carrying plasmid, we performed conjugation experiments using three *S.* Derby strains, five *S.* Enteritidis strains, four *S.* Typhimurium strains, four *armA*-negative *S.* Indiana strains, and three *S.* Indiana strains in which the *armA*-carrying plasmid had been eliminated as recipients and using three *E. coli* J53 strains with different *armA*-carrying plasmid lengths as donors. In total, 57 independent conjugative experiments were performed. The resulting transformants were selected on LB agar with amikacin (30 μg/ml) and streptomycin (4,000 μg/ml), and successful transconjugants were confirmed by real-time PCR. The transfer frequency is expressed as the number of transconjugants per total recipients. The transfer frequency data were analyzed with a multiway analysis of variance using SPSS 19 statistic software.

## Results

### *armA*-Harboring *Salmonella* Emerged in the Diarrheal Outpatients in Shanghai District Around 2011

A total of 45 *armA*-harboring strains were detected in the 15,782 tested *Salmonella* strains. Of these, 13 (positive rate: 0.11%) were from the 12,095 outpatient strains collected in Shanghai (human source), and 32 (1.00%) were from the 3,687 food/environmental isolates.

The first *armA*-harboring strain was found in 2011 (Supplementary Table 3). Except in 2014, two or three *armA*-harboring *Salmonella* strains were identified each year. After the initial appearance of *armA*-harboring *Salmonella* in 2011, the prevalence rate of these strains in community-acquired diarrheal cases in Shanghai was 0.83/1,000 (13/10,234). Of the *armA*-harboring food/environmental-source strains, 22 (positive rate: 0.69%) were from the 3,206 strains collected in Shanghai and 10 (2.07%) were from the 481 strains collected in Guangdong.

To detect other concomitant aminoglycoside-resistant genes in these 45 *armA*-harboring *Salmonella* strains, PCR and qPCR were performed. Of the known aminoglycoside-modifying enzymes genes, *ant(2”)-Ia*, *aph(3’)-Ia*, *aac(3)-Ia*, *aac(3)-IIa*, and *aac(6’)-Ib* were detected. The positive rates of these genes were 31.1% (14/45), 11.1% (5/45), 68.9% (31/45), 8.9% (4/45), and 86.7% (39/45), respectively. The positive rates of *rmtB*, *rmtC*, and *rmtD* were 15.6% (7/45), 2.2% (1/45), and 44.4% (20/45), respectively; in contrast, the *rmtA*, *rmtE*, *rmtF*, *rmtG*, *rmtH*, and *npmA* genes were not detected in any of the isolates.

### The Detected *armA*-Harboring *Salmonella* Strains Show a Highly Skewed Serotype Distribution

Among the 45 *armA*-harboring *Salmonella* strains, nine different serotypes were identified. The serotype distribution was heavily skewed toward serovar Indiana, with 34 strains (75.6%) identified as *S.* Indiana. Other identified serotypes included Thompson (three strains), Kottbus (two strains), and six different serovars with one strain each (Agona, Singapore, Schwerin, Corvallis, Goldcoast, and Infantis).

### The Detected *armA*-Harboring *Salmonella* Strains Were All Multi-Antibiotic Resistant, and Most Carried Extended Spectrum β-Lactamase (ESBL)/Plasmid-Mediated Quinolone Resistance (PMQR) Genes

The results reveal that all these strains were multidrug resistant (MDR), i.e., they exhibited high levels of resistance to more than three classes of antibiotics (Supplementary Table 3). Resistance to amikacin, erythromycin, tobramycin, and gentamicin was observed in all 45 *armA*-harboring *Salmonella* isolates. Additionally, 40 of the strains (including 13 from outpatients and 27 from food/environmental samples) were found to be fluoroquinolone-resistant, and 35 of these fluoroquinolone-resistant strains (including 10 from outpatients and 25 from food/environmental samples) were identified as ESBL producers. In contrast, all 45 *armA*-harboring *Salmonella* strains were susceptible to both imipenem and tigecycline. The ampicillin-resistant rates of the *armA*-harboring *Salmonella* strains isolated from poultry or outpatients were much higher than that of *armA*-harboring *Salmonella* strains isolated from swine (36/37 vs. 2/4).

Among the 45 *armA*-harboring *Salmonella* isolates, the prevalence of ESBL/AmpC genes *bla*_*TEM*__–1B_, *bla*_*CMY*_, *bla*_OXA_, and *bla*_*SHV*__–12_ were 46.7% (21/45), 2.2% (1/45), 68.9% (31/45), and 2.2% (1/45), respectively. The prevalence of *bla*_*CTX–M*_ was 68.9% (31/45). Notably, the extent of resistance to third generation cephalosporin mediated by different *bla*_*CTX–M*_ subtypes is distinct; we found 21 strains with *bla*_*CTX–M–65*_, 6 strains with *bla*_*CTX–M–55*_, 3 strains with *bla*_*CTX–M–3*_, 2 strains with *bla*_*CTX–M–27*_, and 1 strain with *bla*_*CTX–M–14*_. The *bla*_*DHA*_ gene was not detected in these strains (Supplementary Table 3).

The prevalence of PMQR genes *qnrA*, *qnrD*, *qnrS*, *oqxA*, *oqxB*, and *qepA* was 2.2% (1/45), 11.1% (5/45), 15.6% (7/45), 48.9% (22/45), 48.9% (22/45), and 2.2% (1/45), respectively. The *qnrB* and *qnrC* genes were not detected. Additionally, neither the colistin-resistant *mcr-1* gene nor the carbapenem-resistant *bla*_*NDM*_ gene was found to be present in any of the 45 *armA*-harboring *Salmonella* strains.

### The Detected *armA*-Harboring *Salmonella* Strains Exhibited Diverse Genomic Subtyping Patterns

**FIGURE 1:**
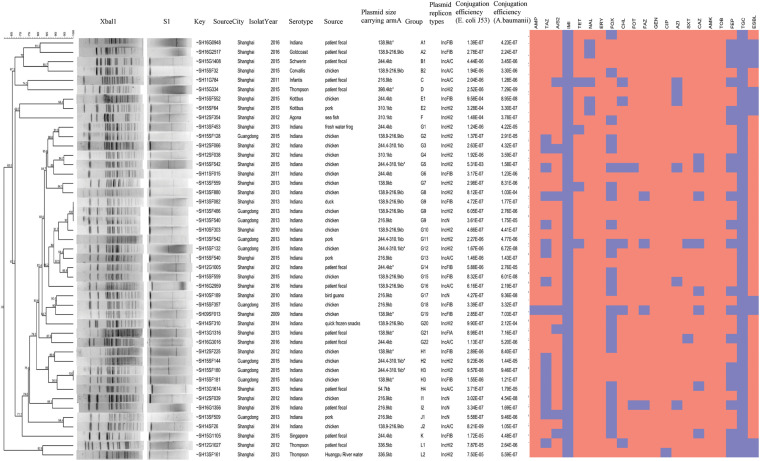
Generated dendrograms showing the cluster analysis of *Xba*I-digested PFGE patterns of the *armA*-harboring *Salmonella* strains isolated in this study.

All 45 *armA*-harboring *Salmonella* strains had different PFGE profiles, which generally had low similarity with one another, suggesting that these isolates are unlikely to have originated from a single clone of *Salmonella*. Only two PFGE clusters were found; one pattern group included strains SH13SF082, SH13SF466, and SH13SF540, which were isolated from food in 2013, and the other included strains SH15SF180 and SH15SF181, which were isolated from food in 2015. The strains in each cluster had indistinguishable PFGE patterns (Figure 1). The PFGE patterns of *S*. Indiana strains isolated from outpatients were more similar to those of the strains isolated from poultry (e.g., SH12G1005 and SH15SF559, SH13G1614 and SH15SF180/SH15SF181, SH12SF039 and SH16G1356) than to those of strains isolated from swine.

### The *armA* Genes in *armA*-Positive *Salmonella* Strains Were Located on Plasmids

Pulsed field gel electrophoresis using *Xba*I and S1 nuclease digestion followed by Southern-hybridization analysis revealed that all 45 isolates, regardless of their conjugative status, carried only a single *armA*-carrying plasmid. Based on the length calculations for the hybridized bands, all the *armA*-carrying plasmids were approximately 54.7–398.4 kb in size.

We performed plasmid elimination on these 45 *armA*-harboring *Salmonella* strains via treatment with SDS and found that all the *armA*-carrying plasmids could be successfully eliminated, i.e., the *armA* gene was not detected in the plasmid-eliminated strains, which supports the conclusion that the *armA* genes in these strains were located only on plasmids. To determine the replicon type(s) of the *armA*-carrying plasmids, replicon-typing PCR, which detects the replicon sequences of plasmid types, was performed. The results show that the *armA*-carrying plasmids included IncHI2 (19 strains, 42.2%), IncFIB (12 strains, 26.7%), IncA/C (8 strains, 17.8%), IncN (5 strains, 11.1%), and IncFIA (1 strain, 2.2%). IncHI2 was the dominant type of *armA*-carrying plasmid in these *Salmonella* strains.

### The *armA*-Carrying Plasmids Were Transferable, and *S*. Indiana Strains Acquired the *armA*-Carrying Plasmids More Easily

**FIGURE 2:**
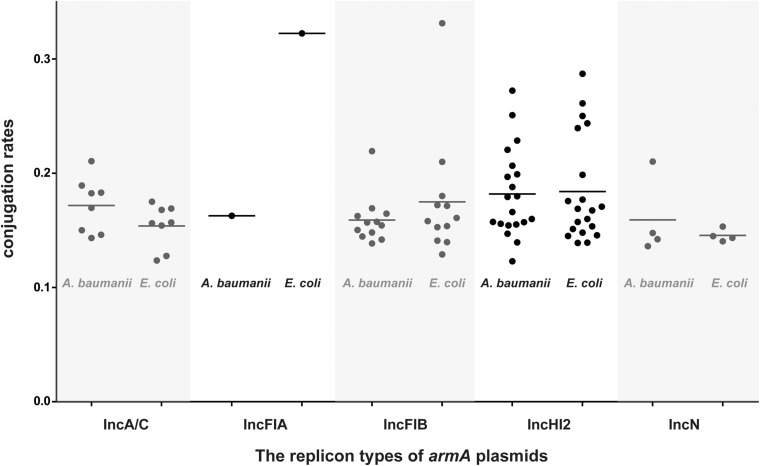
Conjugation rates of the *armA*-harboring plasmids isolated in this study. All 45 detected *armA*-harboring *Salmonella* strains were used as the donors, and either *E. coli* J53 or *A. baumannii* was used as the recipient. Black circles show the 1/abs (log_10_ transformation of the transfer frequency) data.

Conjugation experiments revealed that all 45 *armA*-harboring *Salmonella* isolates were able to transfer their *armA*-carrying plasmids to both *E. coli* J53 and *A. baumannii*, respectively. The transfer frequencies were low, ∼10^–6^ CFU/donor for both *E. coli* J53 and *A. baumannii* (Supplementary Table 2 and Figure 2).

**FIGURE 3:**
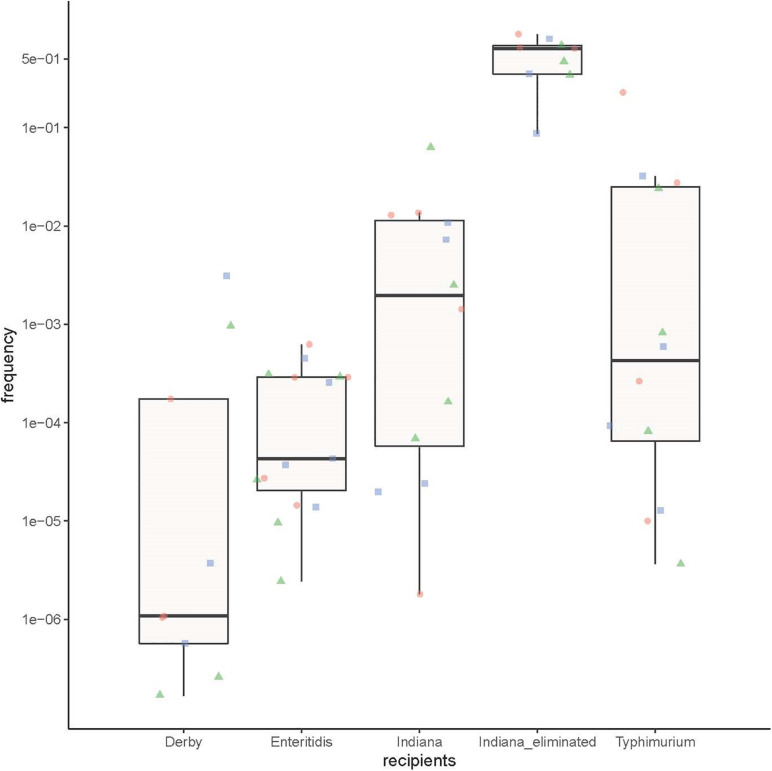
The ability of the different *Salmonella* serotype strains to obtain an *armA*-carrying plasmid. Three *S.* Derby strains, five *S.* Enteritidis strains, four *S.* Typhimurium strains, four naturally *armA*-negative *S.* Indiana strains, and three *S.* Indiana strains in which the *armA*-carrying plasmid was artificially eliminated were used as recipients, and three *E. coli* J53 strains with different lengths of *armA*-carrying plasmid (∼140 kb: green triangles, ∼220 kb: red circles, and ∼300 kb: blue squares) were used as donors.

The transfer frequency data were analyzed by a multiway analysis of variance, and the factors used in this analysis were: five different recipients (*S.* Derby, *S.* Enteritidis, *S.* Typhimurium, *armA*-negative *S.* Indiana, and *S.* Indiana strains in which the *armA*-carrying plasmid was eliminated), and *armA*-carrying plasmids of three different lengths (∼140, ∼220, and ∼300 kb) as donors. There was no significant difference among the different serotypes in their ability to acquire *armA*-carrying plasmids. In contrast, the *S.* Indiana strains in which the *armA*-carrying plasmids were eliminated were able to re-acquire *armA*-carrying plasmids with higher frequencies compared with the other recipients (*p* < 0.01, multi-way ANOVA) (Figure 3).

## Discussion

Aminoglycosides are the antibiotics used most commonly in clinical treatment and veterinary applications. Unfortunately, aminoglycoside use in economically important animals has impelled the emergence and spread of aminoglycoside-resistant bacteria, especially members of the Enterobacteriaceae family. Plasmids carrying 16S rRNA methylase genes, which are responsible for aminoglycoside resistance, have been widely recognized in Enterobacteriaceae family members; however, there are no reports of an epidemiology-based analysis of the *armA* gene prevalence in *Salmonella* spp. isolated from outpatients. Here, we examined samples from a systemic laboratory-based salmonellosis surveillance that was performed yearly from 2005 in diarrheal outpatients in Shanghai; the application of this study design should allow for a reasonable epidemiologic estimate of the prevalence of *armA*-carrying *Salmonella* strains.

The first isolation of an *armA*-harboring *Salmonella* strain occurred in 2011; fortuitously, no increasing trend for *armA* presence was found in the community-acquired infections in Shanghai. As with other clinical Enterobacteriaceae isolates (Samadi et al., 2015), the *armA* prevalence rate in clinical *Salmonella* isolates from the present work was found to remain low over the study period. However, the presence of other aminoglycoside-resistance genes was not assessed, and antimicrobial susceptibility testing was not performed on all the isolated strains from this study, only the strains with *armA*, so strains with other aminoglycoside resistance mechanisms may have remained undetected.

*Salmonella* strains isolated from food and food animal samples were also investigated in our study. These samples were obtained in Shanghai and Guangzhou, and the resulting isolates showed higher *armA*-positive rates compared with the outpatient isolates, suggesting that food and food animals may be a potential threat for the spread of *armA*-mediated aminoglycoside resistance to humans. Although the overall prevalence of strains harboring *armA* was low in this study, all 45 of the *armA*-harboring *Salmonella* isolates were MDR, and 35/45 strains were resistant to both cephalosporins and fluoroquinolone; notably, this indicates a high level of resistance by these strains to the antibiotics that are commonly used in the treatment of salmonellosis patients. The coexistence of the *armA* gene together with ESBL/PMQR genes in the *Salmonella* strains of animal origin may also present a potential risk to human health if the plasmids containing these genes are disseminated via contaminated food. Other studies have similarly found evidence for the coexistence of ESBL/PMQR genes with the *armA* gene in *Salmonella* strains from both food and human sources (Bouzidi et al., 2011; Fang et al., 2019). This situation may pose threats to both food safety and health management owing to two factors: (1) the active horizontal transfer of MDR genes among *Salmonella* and into other Enterobacteriaceae species, and (2) the preservation and possible spread of MDR genes through co-selection with other antibiotics that are commonly used for diarrhea treatment or for improved growth in food-producing animals. The complete sequences could provide more information about the *amrA*-bearing plasmids. Therefore, to provide more characteristic about the *amrA*-positive plasmid, we are looking forward to obtain the complete plasmid sequence base on long-reads sequencing in the future, with the cost of long-reads sequencing decline.

In the present work, we obtained *Salmonella* strains isolated from three different sources (humans, poultry, and swine). The *S*. Indiana strains isolated from outpatients had similar PFGE patterns compared with the strains isolated from poultry, suggesting that *Salmonella* with *armA*-mediated aminoglycoside resistance may be carried by poultry and transmitted from poultry products to humans. Antibiotics are used frequently and extensively in the poultry industry, which has led to the emergence of MDR *Salmonella* (Witte, 1998), and strains of MDR *Salmonella* originating from poultry have the potential to be spread to humans through the food chain (Afema et al., 2016). Poultry and poultry products are recognized as the major vehicles for *Salmonella* transmission to humans (Mezal et al., 2013). In this study, the ampicillin resistance rates of *Salmonella* strains isolated from poultry or humans were much higher than that of strains isolated from swine, which may be related to the excessive use of ampicillin antibiotics in poultry breeding and human medicine (Roth et al., 2019); this finding further supports the idea that the *armA* gene may be transmitted to humans via poultry products.

Pulsed field gel electrophoresis assessment of the 45 *armA*-harboring *Salmonella* strains obtained in this study revealed diverse genetic clones, suggesting that these strains are genetically unrelated and that clonal spread has not been the major mechanism of *armA* gene transmission in *Salmonella*. Thus, compared with the clonal spread of *armA*-harboring *Salmonella*, the transmission of *armA*-carrying plasmids may be more common. Additionally, our results indicate that the *armA* genes in all 45 *Salmonella* isolates were located on plasmids, and plasmids from these strains could be successfully transferred into *E. coli* and *A. baumannii* strains. The *armA*-harboring plasmids comprised five types; the major type, IncHI2, is an MDR plasmid that has been associated with a range of antibiotic resistance genes in *Salmonella* spp. and *E. coli* isolated from humans and food-producing animals (Zhong et al., 2017). The detection of diverse genetic clones and plasmids provides insight into the complicated and likely frequent transmission of *armA*-carrying plasmids among *Salmonella* strains and other Enterobacteriaceae species.

In the present study, 34 of the 45 *armA*-harboring *Salmonella* strains belong to serovar Indiana, raising the question of whether *S*. Indiana is more capable of obtaining these plasmids compared with *Salmonella* strains belonging to other serovars. We compared the plasmid acquisition abilities among *S.* Indiana and some other common *Salmonella* serotypes and found that the group of *armA* plasmid-carrying *S*. Indiana strains from the present study had a greater ability to obtain these plasmids. Interestingly, this level was even higher compared with the tested group of *S*. Indiana strains that lacked *armA*-carrying plasmids, suggesting that a specific genetic characteristic in these *armA*-harboring *S*. Indiana strains is required for the acquisition and maintenance of *armA*-carrying plasmids. In recent years, *S.* Indiana has been frequently isolated from chicken and broilers, as well as from poultry-farming workers, and it has gradually become one of the most common serotypes that cause animal and human salmonellosis (Xia et al., 2009; Lai et al., 2013; Gong et al., 2016). *S*. Indiana isolates that have concurrent resistance to cefotaxime, amikacin, and ciprofloxacin are widespread among chickens in China (Wang et al., 2017b). The MDR *S.* Indiana isolates in China belong to a wide variety of genotypes, suggesting that they probably evolved from many widely dispersed areas rather than from a few more local sources (Luque et al., 2009; Punia et al., 2010; Yang et al., 2010; Lai et al., 2014; Yan et al., 2014; Zhang et al., 2016). Other studies have demonstrated that *S.* Indiana isolates usually carry one or more drug resistance plasmids (Lai et al., 2013; Wang et al., 2016, 2017a), which further supports the important role of *S.* Indiana in public health.

## Conclusion

We conducted a retrospective study using samples collected between 2005 and 2016 from diarrheal outpatients who participated in a continuous *Salmonella* infection surveillance in Shanghai Municipality, China. Our data reveal that, since 2011, the prevalence of *Salmonella* with *armA*-containing plasmids in community-acquired diarrhea cases was low and steady but that all the *armA*-harboring *Salmonella* strains were MDR and were notably resistant to fluoroquinolone and/or extended-spectrum cephalosporins.

The use of aminoglycoside antibiotics is contributing to the rise of MDR *Salmonella*, which poses an increasing public health threat and presents a considerable challenge for the treatment of clinical infections. Future surveillance is necessary to monitor the prevalence and transmission of *armA*-harboring *Salmonella*, especially *S.* Indiana, both in humans and animals and to better understand the potential threat to public health posed by these strains.

## Data Availability Statement

The original contributions presented in the study are included in the article/Supplementary Material, further inquiries can be directed to the corresponding author/s.

## Author Contributions

XL, MZ, and NZ wrote the manuscript. MW, BG, JL, HJ, and WX provided the technical assistance. ZL, HZa, HZo, and ZPL performed the data analysis and prepared the resources. JX, XX, and BK edited the manuscript. All authors read and approved the article.

## Conflict of Interest

The authors declare that the research was conducted in the absence of any commercial or financial relationships that could be construed as a potential conflict of interest.

## Footnotes

1 http://www.ncbi.nlm.nih.gov/BLAST/

## Supplementary Material

Supplementary Table 1 Primers used in this study for polymerase chain reaction.

Supplementary Table 2 The conjugation rates of the *armA*-harboring plasmids.

Supplementary Table 3 Basic information about the 45 armA-harboring *Salmonella* strains identified in this study and their armA-carrying plasmids.
