# Supplementary material for: Prevalence of 16S rRNA Methylation Enzyme Gene armA in Salmonella From Outpatients and Food
Source: Front Microbiol. 2021 May 25;12:663210. doi: 10.3389/fmicb.2021.663210 (PMC8186500; doi:10.3389/fmicb.2021.663210)
Supplement: Supplementary Table 1 — Primers used in this study for polymerase chain reaction. [file Table_1.docx]

Supplementary Table 1. Primers used in this study for polymerase chain reaction.

| 16S RMTase gene | Target gene | Sequences of primers (5' to 3’) | Reference |
| --- | --- | --- | --- |
| rmtA_F | *rmtA* | CTAGCGTCCATCCTTTCCTC | (23) |
| rmtA_R |  | TTTGCTTCCATGCCCTTGCC |  |
| rmtB_F | *rmtB* | CCCAAACAGACCGTAGAGGC |  |
| rmtB_R |  | CTCAAACTCGGCGGGCAAGC |  |
| rmtC_F | *rmtC* | CGAAGAAGTAACAGCCAAAG |  |
| rmtC_R |  | ATCCCAACATCTCTCCCACT |  |
| rmtD_F | *rmtD* | CGGCACGCGATTGGGAAGC |  |
| rmtD_R |  | CGGAAACGATGCGACGAT | (24) |
| rmtE_F | *rmtE* | ATGAATATTGATGAAATGGTTGC |  |
| rmtE_R |  | TGATTGATTTCCTCCGTTTTTG |  |
| rmtF_F | *rmtF* | GCGATACAGAAAACCGAAGG |  |
| rmtF_R |  | ACCAGTCGGCATAGTGCTTT |  |
| rmtG_F | *rmtG* | AAATACCGCGATGTGTGTCC |  |
| rmtG_R |  | ACACGGCATCTGTTTCTTCC |  |
| rmtH_F | *rmtH* | GCTTAAACCCGCTGATGCT |  |
| rmtH_R |  | AAACCAGGTGGCGTAGTGC |  |
| npmA_F | *npmA* | GGAGGGCTATCTAATGTGGT |  |
| npmA_R |  | GCCCAAAGAGAATTAAACTG |  |
| ant(2”)-Ia_F | *ant(2”)-Ia* | GCTTACGTTGTCCCGCATTT | (23) |
| ant(2”)-Ia_R |  | CCTTGGTGATCTCGCCTTTC |  |
| aph(3’)-Ia_F | *aph(3’)-Ia* | CGAGCATCAAATGAAACTGC |  |
| aph(3’)-Ia_R |  | GCGTTGCCAATGATGTTACAG |  |
| aac(3)-Ia_F | *aac(3)-Ia* | GACATAAGCCTGTTCGGTT |  |
| aac(3)-Ia_R |  | CTCCGAACTCACGACCGA |  |
| aac(3)-IIa_F | *aac(3)-IIa* | ATGCATACGCGGAAGGC |  |
| aac(3)-IIa_R |  | TGCTGGCACGATCGGAG |  |
| aac(6’)-Ib_F | *aac(6’)-Ib* | AAGCGTTTTAGCGCAAGAGT |  |
| aac(6’)-Ib_R |  | GCGTGTTTGAACCATGTACA |  |
| bla_DHA__F | *bla_DHA_* | CTGATGAAAAAATCGTTATC | (25) |
| bla_DHA__R |  | ATTCCAGTGCACTCCAAAATA |  |
| bla_TEM__F | *bla_TEM_* | CATTTCCGTGTCGCCCTTATTC |  |
| bla_TEM_ _R |  | CGTTCATCCATAGTTGCCTGAC |  |
| bla_CMY_ _F | *bla_CMY_* | GACAGCCTCTTTCTCCACA |  |
| bla_CMY_ _R |  | TGGAACGAAGGCTACGTA |  |
| bla_OXA_ _F | *bla_OXA_* | GGCACCAGATTCAACTTTCAAG |  |
| bla_OXA_ _R |  | GACCCCAAGTTTCCTGTAAGTG |  |
| bla_CTX_ _F | *bla_CTX_* | TTTGCGATGTGCAGTACCAGTAA |  |
| bla_CTX_ _R |  | CGATATCGTTGGTGGTGCCATA |  |
| bla_SHV_ -F | *bla_SHV_* | TTATCTCCCTGTTAGCCACC |  |
| bla_SHV_ -R |  | GATTTGCTGATTTCGCTCGG | (25) |
| armA-F(qPCR) | *armA* | TCAAAAACCTATACTTTATCGTCGTCTT |  |
| armA-R(qPCR) |  | TATTTTAGATTTTGGTTGTGGCTTCA |  |
| armA-P(qPCR) |  | AACTTCCCAATAATGCTAC |  |
| NDM-F(qPCR) | *bla*NDM | CTTATGCCZZTGCGTTGTCG |  |
| NDM-R(qPCR) |  | CGATCCCAACGGTGATATTGTC |  |
| NDM-P(qPCR) |  | CACAGCCTGCACTTTCGCCGCC |  |
| MCR-F(qPCR) | *mcr* | TCGGCTTTGTGCTGACGAT |  |
| MCR-R(qPCR) |  | AAATCAACACAGGCTTTAGCACATA |  |
| MCR-P(qPCR) |  | CTGTCGTGCTCTTTG |  |
| qnrA_F | *qnrA* | TCAGCAAGAGGATTTCTCA |  |
| qnrA_R |  | GGCAGCACTATTACTCCCA |  |
| qnrB_F | *qnrB* | ATGACGCCATTACTGTATAA |  |
| qnrB_R |  | GATCGCAATGTGTGAAGTTT |  |
| qnrC_F | *qnrC* | GGGTTGTACATTTATTGAATC |  |
| qnrC_R |  | TCCACTTTACGAGGTTCT |  |
| qnrD_F | *qnrD* | CGAGATCAATTTACGGGGAATA |  |
| qnrD_R |  | AACAAGCTGAAGCGCCTG |  |
| qnrS_F | *qnrS* | ACCTTCACCGCTTGCACATT |  |
| qnrS_R |  | CCAGTGCTTCGAGAATCAGT |  |
| qepA_F | *qepA* | CGTGTTGCTGGAGTTCTTC |  |
| qepA_R |  | CTGCAGGTACTGCGTCATG |  |

Supplementary Table 2. The conjugation rates of the *armA*-harbouring plasmids.

| Strains carrying *armA*-plasmids | Transfer frequencies | |
| --- | --- | --- |
|  | *E. coli* J53 | *A. baumanii* |
| SH09SF013 | 2.85E-07 | 7.04E-07 |
| SH10SF189 | 1.07E-07 | 9.36E-08 |
| SH10SF303 | 2.44E-07 | 4.41E-07 |
| SH11G784 | 3.98E-07 | 1.28E-06 |
| SH11SF015 | 6.96E-08 | 1.24E-06 |
| SH12G1005 | 1.47E-06 | 2.76E-05 |
| SH12G1027 | 7.87E-05 | 2.64E-06 |
| SH12SF038 | 1.06E-06 | 3.59E-07 |
| SH12SF039 | 7.54E-08 | 4.54E-08 |
| SH12SF066 | 6.57E-08 | 4.32E-07 |
| SH12SF225 | 2.80E-06 | 8.40E-07 |
| SH12SF354 | 0.000148 | 3.78E-07 |
| SH13G1316 | 0.000791 | 7.16E-07 |
| SH13G1614 | 3.27E-07 | 1.79E-05 |
| SH13SF082 | 4.72E-07 | 1.77E-07 |
| SH13SF161 | 6.67E-05 | 5.59E-07 |
| SH13SF453 | 1.20E-06 | 4.22E-05 |
| SH13SF466 | 4.42E-07 | 2.76E-06 |
| SH13SF509 | 5.58E-07 | 9.46E-06 |
| SH13SF540 | 3.00E-07 | 1.75E-05 |
| SH13SF542 | 2.02E-06 | 4.77E-06 |
| SH13SF559 | 1.76E-07 | 8.31E-06 |
| SH13SF880 | 3.05E-07 | 0.000103 |
| SH14SF26 | 8.21E-09 | 1.05E-07 |
| SH14SF310 | 1.29E-07 | 0.000213 |
| SH15G1105 | 1.72E-05 | 4.48E-07 |
| SH15G1408 | 1.11E-06 | 3.45E-06 |
| SH15G334 | 2.24E-06 | 7.29E-09 |
| SH15SF128 | 1.37E-07 | 2.91E-05 |
| SH15SF132 | 1.39E-06 | 6.72E-08 |
| SH15SF144 | 9.23E-06 | 1.45E-05 |
| SH15SF180 | 6.41E-08 | 9.46E-07 |
| SH15SF181 | 1.55E-06 | 1.21E-07 |
| SH15SF32 | 1.94E-06 | 3.30E-06 |
| SH15SF357 | 3.12E-07 | 3.32E-07 |
| SH15SF540 | 1.22E-06 | 1.43E-07 |
| SH15SF542 | 0.0001 | 1.58E-07 |
| SH15SF552 | 0.000958 | 8.95E-08 |
| SH15SF559 | 6.08E-07 | 6.01E-08 |
| SH15SF64 | 0.000328 | 3.30E-07 |
| SH16G0948 | 1.74E-08 | 4.23E-07 |
| SH16G1356 | 1.27E-07 | 1.69E-07 |
| SH16G2517 | 8.06E-08 | 2.24E-07 |
| SH16G2959 | 4.22E-07 | 2.19E-07 |
| SH16G3016 | 1.46E-08 | 5.20E-06 |

**SUPPLEMEBTARY TABLE 3** Basic information about the 45 armA-harboring Salmonella strains identified in this study and their armA-carrying plasmids

| Strains | Sampling year | Serotypes | location | source | The Aminoglycosides modifying enzyme genes | other 16S rRNA methylase genes | PMQR genes | ESBL/AmpC genes | Antibiotic resistance | ESBL-producing strains | armA plasmid type | armA-plasmid approximate length (kb) |
| --- | --- | --- | --- | --- | --- | --- | --- | --- | --- | --- | --- | --- |
| SH09SF013 | 2009 | Indiana | Shanghai | chicken | *aac(6)-Ib* | *rmtB*, *rmtD* | NF | NF | TET, NAL, ERY, FOT, GEN, CIP, AZI, SXT, AMK, TOB | N | IncFIB | ~138.9 |
| SH10SF189 | 2010 | Indiana | Shanghai | bird guano | *aac(3)-Ia*, *aac(6)-Ib* | NF | NF | *bla*_OXA_, *bla*_CTX-M-65_ | AMP, TAZ, A/S2, TET, NAL, ERY, CHL, FOT, FAZ, GEN, CIP, AZI, SXT, AMK, TOB | Y | IncN | ~216.9 |
| SH10SF303 | 2010 | Indiana | Shanghai | chicken | *aac(3)-Ia*, *aac(6)-Ib* | NF | *qnrD*, *oqxA*, *oqxB* | *bla*_TEM_ _-1B_, *bla*_OXA_, *bla*_CTX-M-65_ | AMP, A/S2, TET, NAL, ERY, CHL, FOT, FAZ, GEN, CIP, AZI, SXT, CAZ, AMK, TOB | Y | IncHI2 | 138.9-216.9 |
| SH11G784 | 2011 | Infantis | Shanghai | patient fecal | *ant(2")-Ia*, *aac(3)-Ia*, *aac(6)-Ib* | NF | NF | *bla*_CTX-M-14_ | AMP, TAZ, A/S2, TET, NAL, ERY, CHL, FOT, FAZ, GEN, CIP, AZI, SXT, CAZ, AMK, TOB, FEP | Y | IncA/C | ~216.9 |
| SH11SF015 | 2011 | Indiana | Shanghai | chicken | *aac(3)-Ia*, *aac(6)-Ib* | NF | *oqxA*, *oqxB* | *bla*_OXA_ | AMP, A/S2, TET, NAL, ERY, FAZ, GEN, CIP, SXT, AMK, TOB | Y | IncFIB | ~244.4 |
| SH12G1005 | 2012 | Indiana | Shanghai | patient fecal | *ant(2")-Ia*, *aac(3)-Iaaph(3)-Ia*, *aac(6)-Ib* | NF | *qnrD*, *oqxA*, *oqxB* | *bla*_TEM-1B_, *bla*_CTX-M-65_ | AMP, TAZ, A/S2, TET, NAL, ERY, FOX, CHL, FOT, FAZ, GEN, CIP, AZI, SXT, CAZ, AMK, TOB, FEP | Y | IncFIB | ~138.9 |
| SH12G1027 | 2012 | Thompson | Shanghai | patient fecal | *aac(3)-Ia* | NF | NF | *bla*_CTX-M-3_ | AMP, A/S2, TET, NAL, ERY, FO, XFOT, FAZ, GEN, CIP, AZI, SXT, CAZ, AMK, TOB | N | IncHI2 | ~336.5 |
| SH12SF038 | 2012 | Indiana | Shanghai | chicken | *aac(3)-Ia*, *aac(6)-Ib* | NF | NF | *bla*_OXA_, *bla*_CTX-M-65_ | AMP, TET, NAL, ERY, CHL, FOT, FAZ, GEN, CIP, AZI, SXT, CAZ, AMK, TOB, FEP | Y | IncHI2 | ~310.1 |
| SH12SF039 | 2012 | Indiana | Shanghai | chicken | *aac(3)-Ia*, *aac(6)-Ib* | *rmtD* | NF | *bla*_OXA_, *bla*_CTX-M-65_ | AMP, A/S2, TET, NAL, ER, YCHL, FOT, FAZ, GEN, CIP, AZI, SXT, CAZ, AMK, TOB, FEP | Y | IncN | ~216.9 |
| SH12SF066 | 2012 | Indiana | Shanghai | chicken | *aac(3)-Ia*, *aac(6)-Ib* | *rmtD* | *oqxA*, *oqxB* | *bla*_OXA_, *bla*_CTX-M-65_ | AMP, A/S2, TET, NAL, ERY, CHL, FOT, FAZ, GEN, CIP, AZI, SXT, CAZ, AMK, TOB, FEP | Y | IncHI2 | 244.4-310.1 |
| SH12SF225 | 2012 | Indiana | Shanghai | chicken | *aac(3)-Ia*, *aac(6)-Ib* | NF | *oqxA*, *oqxB* | *bla*_TEM-1B_, *bla*_OXA_, *bla*_CTX-M-65_ | AMP, TAZ, A/S2, TET, NAL, ERY, CHL, FOT, FAZ, GEN, CIP, AZI, SXT, CAZ, AMK, TOB, FEP | Y | IncFIB | ~138.9 |
| SH12SF354 | 2012 | Agona | Shanghai | sea fish | *aac(3)-Ia*, *aac(6)-Ib* | NF | *qnrD* | *bla*_TEM-1B_, *bla*_CTX-M-3_ | AMP, TAZ, A/S2, TET, ERY, FOX, CHL, FOT, FAZ, GEN, CIP, AZI, SXT, CAZ, AMK, TOB | N | IncHI2 | ~310.1 |
| SH13G1316 | 2013 | Indiana | Shanghai | patient fecal | *ant(2")-Ia*, *aac(3)-Ia*, *aph(3)-Ia*, *aac(6)-Ib* | *rmtB*, *rmtD* | NF | *bla*_TEM-1B_, *bla*_OXA_, *bla*_CTX-M-27_ | AMP, TAZ, TET, NAL, ERY, CHL, FOT, FAZ, GEN, CIP, AZI, SXT, CAZ, AMK, TOB, FEP | Y | IncFIA | ~138.9 |
| SH13G1614 | 2013 | Indiana | Shanghai | patient fecal | *aac(3)-Ia*, *aac(6)-Ib* | *rmtD* | *oqxA*, *oqxB* | *bla*_TEM-1B_, *bla*_OXA_, *bla*_CTX-M-65_ | AMP, A/S2, TET, NAL, ERY, CHL, FOT, FAZ, GEN, CIP, AZI, SXT, AMK, TOB | Y | IncA/C | ~54.7 |
| SH13SF082 | 2013 | Indiana | Shanghai | duck | *aac(3)-Ia*, *aac(6)-Ib* | NF | NF | *bla*_OXA_ | AMP, TET, NAL, ERY, CHL, FOT, GEN, CIP, AZI, SXT, AMK, TOB | Y | IncFIB | 138.9-216.9 |
| SH13SF161 | 2013 | Thompson | Shanghai | Huangpu River water | *aac(3)-Ia* | NF | *qnrD* | *bla*_TEM-1B_, *bla*_CTX-M-3_ | AMP, TAZ, A/S2, TET, NAL, ERY, FOX, CHL, FOT, FAZ, GEN, AZI, SXT, CAZ, AMK, TOB | N | IncHI2 | ~336.5 |
| SH13SF453 | 2013 | Indiana | Shanghai | fresh water frog | *aac(3)-Ia*, *aac(6)-Ib* | *rmtB*, *rmtD* | *oqxA*, *oqxB* | *bla*_TEM-1B_, *bla*_OXA_, *bla*_CTX-M-65_ | AMP, TAZ, A/S2, TET, NAL, ERY, CHL, FOT, FAZ, GEN, CIP, AZI, SXT, CAZ, AMK, TOB | Y | IncHI2 | ~244.4 |
| SH13SF466 | 2013 | Indiana | Guangdong | chicken | *aac(3)-Ia*, *aac(6)-Ib* | *rmtD* | NF | *bla*_TEM-1B_, *bla*_OXA_, *bla*_CTX-M-65_ | AMP, TAZ, A/S2, TET, NAL, ERY, FOX, CHL, FOT, FAZ, GEN, CIP, AZI, SXT, CAZ, AMK, TOB, FEP | Y | IncHI2 | 138.9-216.9 |
| SH13SF509 | 2013 | Indiana | Guangdong | pork | *aac(3)-Ia*, *aac(6)-Ib* | *rmtB*, *rmtD* | *oqxA*, *oqxB* | *bla*_TEM-1B_, *bla*_OXA_, *bla*_CTX-M-65_ | TET, NAL, ERY, CHL, FOT, FAZ, GEN, CIP, AZI, SXT, CAZ, AMK, TOB, FEP | Y | IncHI2 | ~216.9 |
| SH13SF540 | 2013 | Indiana | Guangdong | chicken | *aac(3)-Ia*, *aac(6)-Ib* | *rmtD* | *qnrS*, *oqxA* | *bla*_TEM-1B_, *bla*_OXA_, *bla*_CTX-M-65_ | AMP, TAZ, A/S2, TET, NAL, ERY, FOX, CHL, FOT, FAZ, GEN, CIP, AZI, SXT, CAZ, AMK, TOB, FEP | Y | IncN | ~216.9 |
| SH13SF542 | 2013 | Indiana | Guangdong | pork | *aac(3)-Ia*, *aac(6)-Ib* | NF | *oqxA*, *oqxB* | *bla*_TEM-1B_, *bla*_OXA_, *bla*_CTX-M-65_ | AMP, TAZ, A/S2, TET, NAL, ERY, FOX, CHL, FOT, FAZ, GEN, CIP, AZI, SXT, CAZ, AMK, TOB, FEP | Y | IncHI2 | ~244.4 |
| SH13SF559 | 2013 | Indiana | Shanghai | chicken | *aac(3)-Ia*, *aac(6)-Ib* | *rmtD* | *oqxA,* *oqxB* | *bla*_TEM-1B_, *bla*_OXA_, *bla*_CTX-M-65_ | AMP, TAZ, A/S2, TET, NAL, ERY, FOX, CHL, FOT, FAZ, GEN, CIP, AZI, SXT, CAZ, AMK, TOB, FEP | Y | IncHI2 | ~138.9 |
| SH13SF880 | 2013 | Indiana | Shanghai | chicken | *aac(6)-Ib* | NF | *oqxA*, *oqxB* | *bla*_OXA_, *bla*_CTX-M-65_ | AMP, TAZ, A/S2, NAL, ERY, CHL, FOT, FAZ, GEN, CIP, AZI, SXT, CAZ, AMK, TOB, FEP | Y | IncHI2 | 138.9-216.9 |
| SH14SF26 | 2014 | Indiana | Shanghai | chicken | *ant(2")-Ia*, *aac(3)-Ia*, *aac(6)-Ib* | *rmtD* | NF | *bla*_TEM-1B_, *bla*_OXA_, *bla*_CTX-M-65_ | AMP, TAZ, A/S2, TET, NAL, ER, YCHL, FOT, FAZ, GEN, CIP, AZI, SXT, CAZ, AMK, TOB, FEP | Y | IncA/C | 138.9-216.9 |
| SH14SF310 | 2014 | Indiana | Shanghai | quick frozen snacks | *aac(3)-Ia*, *aac(6)-Ib* | NF | *oqxA*, *oqxB* | *bla*_OXA_, *bla*_CTX-M-65_ | AMP, TAZ, A/S2, TET, NAL, ERY, CHL, FOT, FAZ, GEN, CIP, AZI, SXT, CAZ, AMK, TOB, FEP | Y | IncHI2 | 138.9-216.9 |
| SH15G1105 | 2015 | Singapore | Shanghai | patient fecal | *ant(2")-Ia*, *aac(3)-IIa*, *aac(6)-Ib* | NF | *qnrA* | *bla*_TEM-1B_, *bla*_OXA_ | AMP, TAZ, TET, NAL, ERY, CHL, FOT, FAZ, GEN, CIP, AZI, SXT, AMK, TOB | Y | IncFIB | ~244.4 |
| SH15G1408 | 2015 | Schwerin | Shanghai | patient fecal | *ant(2")-Ia*, *aac(3)-IIa*, *aph(3)-Ia*, *aac(6)-Ib* | NF | *qnrS*, *qepA* | *bla*_CMY_, *bla*_OXA_ | AMP, TAZ, A/S2, TET, ERY, FOX, CHL, FOT, FAZ, GEN, CIP, AZI, SXT, CAZ, AMK, TOB | N | IncA/C | ~244.4 |
| SH15G334 | 2015 | Thompson | Shanghai | patient fecal | *aac(3)-Ia* | NF | *qnrD*, *oqxB* | *bla*_TEM-1B_ | AMP, TAZ, ERY, FOX, FOT, FAZ, GEN, CIP, SXT, CAZ, AMK, TOB | Y | IncHI2 | ~398.4 |
| SH15SF128 | 2015 | Indiana | Guangdong | chicken | *aac(3)-Ia*, *aac(6)-Ib* | *rmtD* | NF | *bla*_TEM-1B_, *bla*_OXA_, *bla*_CTX-M-65_ | AMP, TAZ, A/S2, NAL, ERY, FOX, CHL, FOT, FAZ, GEN, CIP, AZI, SXT, CAZ, AMK, TOB, FEP | Y | IncHI2 | 138.9-216.9 |
| SH15SF132 | 2015 | Indiana | Guangdong | chicken | *aac(6)-Ib* | NF | NF | *bla*_OXA_ | AMP, A/S2, NAL, ERY, FOT, GEN, CIP, AZI, AMK, TOB | Y | IncHI2 | 244.4-310.1 |
| SH15SF144 | 2015 | Indiana | Guangdong | chicken | *ant(2")-Ia*, *aac(3)-Ia*, *aac(6)-Ib* | *rmtD* | *oqxA*, *oqxB* | *bla*_TEM-1B_, *bla*_OXA_, *bla*_CTX-M-65_ | AMP, A/S2, TET, NAL, ERY, FOX, CHL, FOT, FAZ, GEN, CIP, AZI, SXT, CAZ, AMK, TOB, FEP | Y | IncHI2 | 244.4-310.1 |
| SH15SF180 | 2015 | Indiana | Guangdong | chicken | *aac(3)-Ia*, *aac(6)-Ib* | *rmtB*, *rmtD* | *oqxA*, *oqxB* | *bla*_TEM-1B_, *bla*_OXA_, *bla*_CTX-M-65_ | AMP, A/S2, TET, NAL, ERY, FOX, CHL, FOT, FAZ, GEN, CIP, AZI, SXT, CAZ, AMK, TOB | Y | IncHI2 | 244.4-310.1 |
| SH15SF181 | 2015 | Indiana | Guangdong | chicken | *aac(3)-Ia*, *aac(6)-Ib* | *rmtD* | *oqxA*, *oqxB* | *bla*_OXA_, *bla*_CTX-M-65_ | AMP, A/S2, TET, NAL, ERY, FOX, CHL, FOT, FAZ, GEN, CIP, AZI, SXT, CAZ, AMK, TOB | Y | IncFIB | ~138.9 |
| SH15SF32 | 2015 | Corvallis | Shanghai | chicken | *ant(2")-Ia*, *aac(3)-IIa*, *aph(3)-Ia*, *aac(6)-Ib* | NF | NF | *bla*_OXA_ | AMP, TAZ, A/S2, TET, NAL, ERY, FOX, CHL, FOT, FAZ, GEN, CIP, AZI, SXT, CAZ, AMK, TOB | N | IncA/C | 138.9-216.9 |
| SH15SF357 | 2015 | Indiana | Guangdong | chicken | *ant(2")-Ia*, *aac(6)-Ib* | *rmtB*, *rmtD* | *oqxA*, *oqxB* | *bla*_TEM-1B_, *bla*_OXA_, *bla*_CTX-M-55_ | AMP, TAZ, A/S2, TET, NAL, ERY, FOX, CHL, FOT, FAZ, GEN, CIP, AZI, SXT, CAZ, AMK, TOB, FEP | N | IncFIB | ~216.9 |
| SH15SF540 | 2015 | Indiana | Shanghai | pork | *ant(2")-Ia*, *aac(6)-Ib* | *rmtD* | *oqxA*, *oqxB* | *bla*_OXA_, *bla*_CTX-M-55_ | AMP, A/S2, TET, NAL, ERY, CHL, FOT, FAZ, GEN, CIP, AZI, SXT, CAZ, AMK, TOB, FEP | Y | IncA/C | ~216.9 |
| SH15SF542 | 2015 | Indiana | Shanghai | chicken | *aac(3)-Ia*, *aac(6)-Ib* | NF | *oqxA*, *oqxB* | *bla*_OXA_ | AMP, TAZ, A/S2, TET, NAL, ERY, FOX, CHL, FOT, FAZ, GEN, CIP, AZI, SXT, AMK, TOB | Y | IncHI2 | 244.4-310.1 |
| SH15SF552 | 2015 | Kottbus | Shanghai | chicken | *aac(3)-Ia*, *aac(6)-Ib* | *rmtD* | *qnrS* | *bla*_CTX-M-55_ | AMP, TAZ, A/S2, TET, NAL, ERY, FOX, CHL, FOT, FAZ, GEN, CIP, SXT, CAZ, AMK, TOB, FEP | N | IncFIB | ~244.4 |
| SH15SF559 | 2015 | Indiana | Shanghai | chicken | *ant(2")-Ia*, *aac(3)-Ia*, *aac(6)-Ib* | *rmtD* | *oqxA*, *oqxB* | *bla*_OXA_, *bla*_CTX-M-55_ | AMP, TAZ, A/S2, TET, NAL, ERY, CHL, FOT, FAZ, GEN, CIP, AZI, SXT, CAZ, AMK, TOB, FEP | Y | IncFIB | 138.9-216.9 |
| SH15SF64 | 2015 | Kottbus | Shanghai | pork | *aac(3)-Ia*, *aac(6)-Ib* | *rmtB* | *qnrS* | *bla*_CTX-M-55_ | TAZ, A/S2, TET, ERY, FOX, FOT, FAZ, GEN, CIP, SXT, CAZ, AMK, TOB, FEP | N | IncHI2 | ~310.1 |
| SH16G0948 | 2016 | Indiana | Shanghai | patient fecal | *aac(6)-Ib* | NF | *oqxA*, *oqxB* | *bla*_CTX-M-55_ | AMP, TAZ, A/S2, TET, NAL, ERY, CHL, FOT, FAZ, GEN, CIP, AZI, SXT, CAZ, AMK, TOB, FEP | Y | IncFIB | ~138.9 |
| SH16G1356 | 2016 | Indiana | Shanghai | patient fecal | NF | *rmtC* | *qnrS*, *oqxA*, oqxB | *bla*_SHV-12_ | AMP, A/S2, TET, NAL, ERY, CHL, GEN, CIP, SXT, CAZ, AMK, TOB | Y | IncN | ~216.9 |
| SH16G2517 | 2016 | Goldcoast | Shanghai | patient fecal | *ant(2")-Ia*, *aac(3)-IIa*, *aph(3)-Ia*, *aac(6)-Ib* | *rmtD* | *qnrS* | *bla*_TEM-1B_ | AMP, TAZ, A/S2, TET, ERY, FOX, CHL, FOT, FAZ, GEN, CIP, AZI, SXT, CAZ, AMK, TOB | N | IncFIB | 138.9-216.9 |
| SH16G2959 | 2016 | Indiana | Shanghai | patient fecal | *ant(2")-Ia*, *aac(6)-Ib* | NF | NF | *bla*_CTX-M-27_ | AMP, A/S2, TET, NAL, ERY, CHL, FOT, FAZ, GEN, CIP, SXT, CAZ, AMK, TOB, FEP | Y | IncA/C | 138.9-216.9 |
| SH16G3016 | 2016 | Indiana | Shanghai | patient fecal | *ant(2")-Ia* | NF | *qnrS* | NF | AMP, TAZ, A/S2, TET, NAL, ERY, CHL, FOT, FAZ, GEN, CIP, AZICAZ, AMK, TOB | Y | IncA/C | ~244.4 |
